# Confirmation of Ogden syndrome as an X‐linked recessive fatal disorder due to a recurrent NAA10 variant and review of the literature

**DOI:** 10.1002/ajmg.a.62351

**Published:** 2021-06-01

**Authors:** Laura Gogoll, Katharina Steindl, Pascal Joset, Markus Zweier, Alessandra Baumer, Christina Gerth‐Kahlert, Boris Tutschek, Anita Rauch

**Affiliations:** ^1^ Institute of Medical Genetics, University of Zurich Schlieren Switzerland; ^2^ Division of Ophthalmology University Hospital Zurich Zurich Switzerland; ^3^ Prenatal Zürich Zürich Switzerland; ^4^ Medical Faculty Heinrich Heine University Düsseldorf Germany; ^5^ University Children's Hospital Zurich Switzerland

**Keywords:** NAA10, NatA, N‐terminal acetylation, Ogden syndrome, XLID

## Abstract

Ogden syndrome is a rare lethal X‐linked recessive disorder caused by a recurrent missense variant (Ser37Pro) in the NAA10 gene, encoding the catalytic subunit of the N‐terminal acetyltransferase A complex (NatA). So far eight boys of two different families have been described in the literature, all presenting the distinctive and recognizable phenotype, which includes mostly postnatal growth retardation, global severe developmental delay, characteristic craniofacial features, and structural cardiac anomalies and/or arrhythmias. Here, we report the ninth case of Ogden syndrome with an independent recurrence of the Ser37Pro variant. We were able to follow the clinical course of the affected boy and delineate the evolving phenotype from his birth until his unfortunate death at 7 months. We could confirm the associated phenotype as well as the natural history of this severe disease. By describing new presenting features, we are further expanding the clinical spectrum associated with Ogden syndrome and review other phenotypes associated with NAA10 variants.

## 1 INTRODUCTION

Ogden syndrome, was named after the hometown of the first family observed by John M. Opitz with an X‐linked recessive disorder characterized by early lethality due to structural cardiac anomalies and/or arrhythmias, severe global developmental delay, postnatal growth retardation, and prematurely aged appearance with reduced subcutaneous adipose tissue and redundant skin (Lyon, 2011; Maffly, 2012; Rope et al., 2011; Wu & Lyon, 2018). In 2011, it was identified as the first human genetic disorder associated with a variant in an N‐terminal acetyltransferase (NAT) (Rope et al., 2011). So far, only the original eight affected males from two different families are described in the literature, all harboring the same missense variant, a Ser37Pro, in the gene encoding NAA10, the catalytic subunit of the N‐terminal acetyltransferase A complex (NatA), the major human NAT involved in the co‐translational acetylation of proteins (Arnesen et al., 2005; Rope et al., 2011). Nevertheless, further phenotypes in males and females have been described with other pathogenic variants resulting in the suggestion of the broader term *NAA10‐*related syndrome (Wu & Lyon, 2018). We now report an independent recurrence of the Ser37Pro variant further supporting the initially described genotype–phenotype relation with the Ogden syndrome clinical course and also expanding its clinical signs.

## 2 CLINICAL REPORT

**FIGURE 1:**
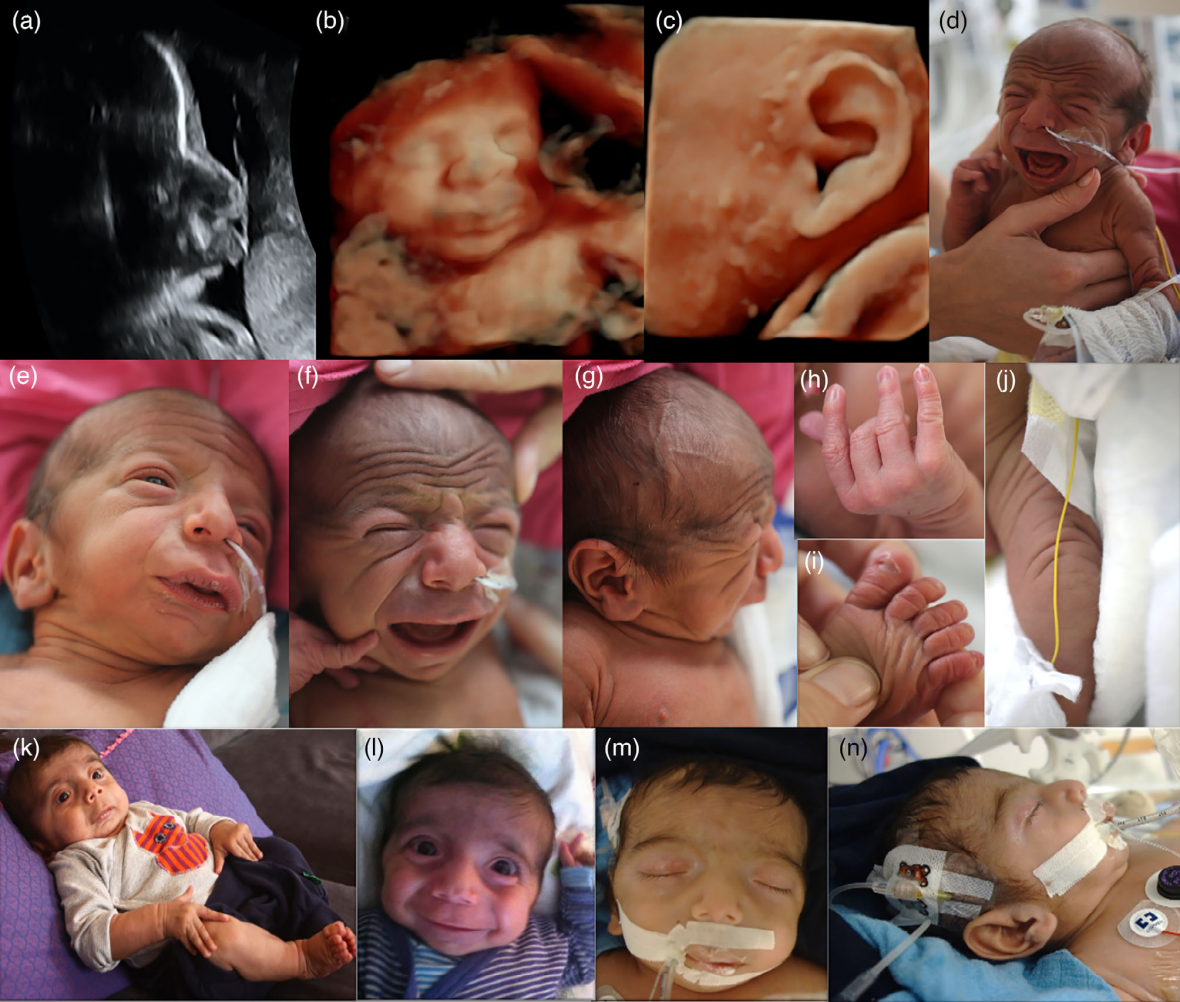
Prenatal ultrasound scans (a‐c) and pictures of our patient at 3 days (d–j), 4 (k and l), and 6 months (m–n) showing the facial apprearance and illustrating the changing phenotype. Note the facial wrinkling, proptosis, hypertelorism, wide nasal bridge, prominent nose, short columella, deep philtrum, wide mouth, everted lower lip vermilion, and microretrognathia. The ears are large, low‐set, malformed, and posteriorly rotated. The fingers are long with mild camptodactyly. Long toes with broad halluces, sandal gap. Note the overall wrinkled loose skin with decrease of subcutaneous fat. Some of the facial features such as apparent hypertelorism, broad nasal root, prominent nose with wide nares and mouth, microretrognathia are already recognizable on prenatal 3D ultrasound scans

**FIGURE 2:**
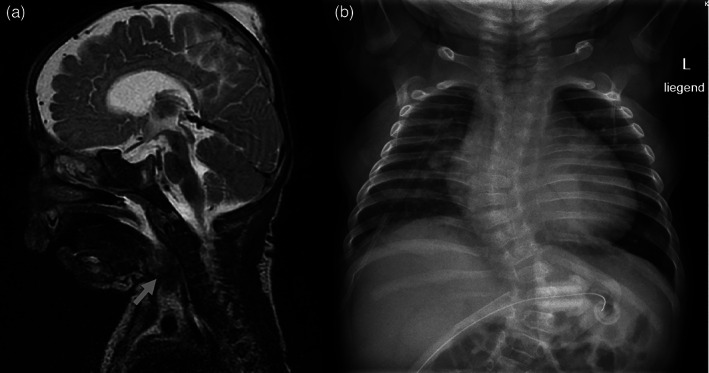
(a) Magnetic resonance imaging of the brain revealed intracranially wide inner and outer cerebrospinal fluid spaces and a very narrow corpus callosum. Note the increased fatty tissue in the epipharynx (grey arrow). (b) X‐ray of the chest showing cardiomegaly, scoliosis and paravertebral left residual consolidation with positive air bronchogram

This male infant was born to healthy, non‐consanguineous parents of German and Polish origin. He was the first child of the 40‐year‐old mother, who had one miscarriage before. The mother had no obvious anomalies, has a high educational attainment (University degrees in economics and psychology), and runs her own business. Paternal age at birth was 57 and his history was unremarkable. The pregnancy was complicated by intrauterine growth retardation (IUGR). An abnormal cardiotocography (CTG) lead to the induction of labor at gestational week 37. His birth weight was 2580 g (−1SD), his birth length was 43 cm (−3SD), and his occipital frontal circumference measured 32.5 cm (−1SD). Apgar scores were 9/9/10. The neonatal examination revealed severe muscular hypotonia and a number of distinctive morphologic signs, in particular the progeroid facial appearance. After 48 h, he was transferred to the neonatal intensive care unit (NICU) because of respiratory distress, feeding difficulties, and hyperbilirubinemia. Clinical genetic evaluation of the 3‐day‐old boy showed facial wrinkling and hirsutism, extension of hair growth on temples to lateral eyebrow, prominent eyes, short and down‐slanting palpebral fissures, apparent hypertelorism, wide nasal bridge, prominent nose, short columella, wide philtrum and mouth, high, narrow palate, thick lingual frenulum, gingival overgrowth, thin upper lip vermilion in frontal view and microretrognathia. The ears were large, low‐set, posteriorly rotated and dysplastic with prominent crus of helix and a large earlobe. In addition, a stenosis of the external auditory canals was observed and he failed the otoacoustic emissions screening. He had a low posterior hair line and a short neck. Nipples were hypoplastic and wide‐spaced. The fingers were long, the thumbs adducted. Mild camptodactyly and neonatally prominent fingertip pads were noticed. His toes were long, halluces were broad with a sandal gap. He had generalized hirsutism. The overall wrinkled and redundant skin with reduced subcutaneous adipose tissue resulted in a prematurely aged appearance (see Figure 1). The echocardiography revealed an atrial septal aneurysm, a persistent foramen ovale, and a bicuspid aortic valve. The chest X‐ray demonstrated cardiomegaly and scoliosis (see Figure 2). The eye examination showed persistent pupillary membranes, an enlarged and oval‐shaped optic disc anomaly and peripheral retinal hyperpigmentation in both eyes. An ultrasound of the hip and the groin confirmed congenital bilateral hip dislocation and unilateral acetabular dysplasia. The abdominal ultrasound was normal. His clinical course was complicated by failure to thrive, frequent desaturations, and hypoglycemia, requiring support with tube feeding, high flow therapy and supplemental nutrition. A diagnosis of Donohue syndrome was considered but insulin levels were normal. After he spent approximately 4 weeks in NICU, tube feeding and monitoring of his oxygen saturation were continued at home. At 6 weeks of age he presented again to the pediatric emergency care with an upper respiratory infection. His weight was 3.05 kg (−2SD). He was discharged from the hospital the same day. At the 9‐weeks follow‐up, his weight was 3.66 kg (−3SD) and his head circumference was 36 cm (−4SD). No further hypoglycemia was noted. Elective hospital admission for percutaneous endoscopic gastrostomy was conducted at the age of 15 weeks. The Cormnack Lehane score III predicted a difficult airway management due to a bulging mass in the epipharynx and fiberoptic intubation was performed. After the intervention he suffered from apneic episodes which lead to an urgent transfer to the NICU. In the following days he suffered from a number of serious complications, including pneumothorax, and several infections (i.e., pneumonia, urinary tract infection, and gastroenteritis). In addition, he developed epileptic seizures, the EEG showed pathologic monomorphic patterns with absence of epileptiform activity. Brain imaging by magnetic resonance imaging (MRI) was performed and revealed enlarged ventricles, a slender corpus callosum, a small pituitary gland, and white matter reduction. The MRI confirmed further the previously noticed craniofacial traits of the viscerocranium, that is, hypertelorism, retrognathia, and deviated nasal septum. In addition, the MRI revealed an adipose tissue mass (DD: teratoma) compressing the epipharynx (see Figure 2). Unfortunately, no histological analysis of this tissue mass was undertaken. The boy underwent extubation after 10 days at the NICU and needed further assisted respiratory ventilation (CPAP‐therapy) for additional 10 days. Due to the complications and the intermittent desaturations, he was hospitalized for almost 5 weeks. The following weeks his general condition fortunately ameliorated; he started to interact with his environment and was able to fixate objects or persons. The severe truncal hypotonia improved under physiotherapy and craniosacral therapy. Surprisingly he showed a remarkable change in the phenotype. He appeared less aged and the facial wrinkling was less prominent, but no Holter or telemetry‐monitoring was done. His weight, length and head circumference remained, however, below the third centile at 4 months of age. Following the molecular diagnosis of Ogden syndrome, a standard electrocardiogram was performed at the ages of 2 weeks and 4 months which revealed no abnormalities. At 6 months of age he was hospitalized a third time with respiratory distress, severe coughing, and vomiting. His general condition was slightly reduced, but he did not appear to be very sick. Initially the respiratory condition could be stabilized, however an increasing agitation and irritability made the respiratory support difficult. Overnight his condition deteriorated rapidly. He was unable to maintain adequate ventilation and displayed agonal respiration and severe desaturations (O2 level: 45%). A high flow ventilation was initiated, and the sedation was augmented by a subcutaneous morphine pump. Because he did not respond to morphine, midazolam was administered. He became more and more agitated and did not tolerate the ventilation. Moreover, he developed two generalized tonic seizures. He developed increased respiratory distress resulting in respiratory acidosis and respiratory failure. Chest X‐rays revealed cardiomegaly, scoliosis, and paravertebral residual consolidation with positive air bronchogram on the left side (see Figure 2), but neither high‐resolution lung CT‐imaging nor lung biopsy was performed. As his condition progressively worsened, his parents chose to stop all life sustaining procedures. He died at the age of 7 months, probably due to complications of an obstructive bronchitis, but no autopsy was undertaken.

## 3 GENETIC STUDIES

**FIGURE 3:**
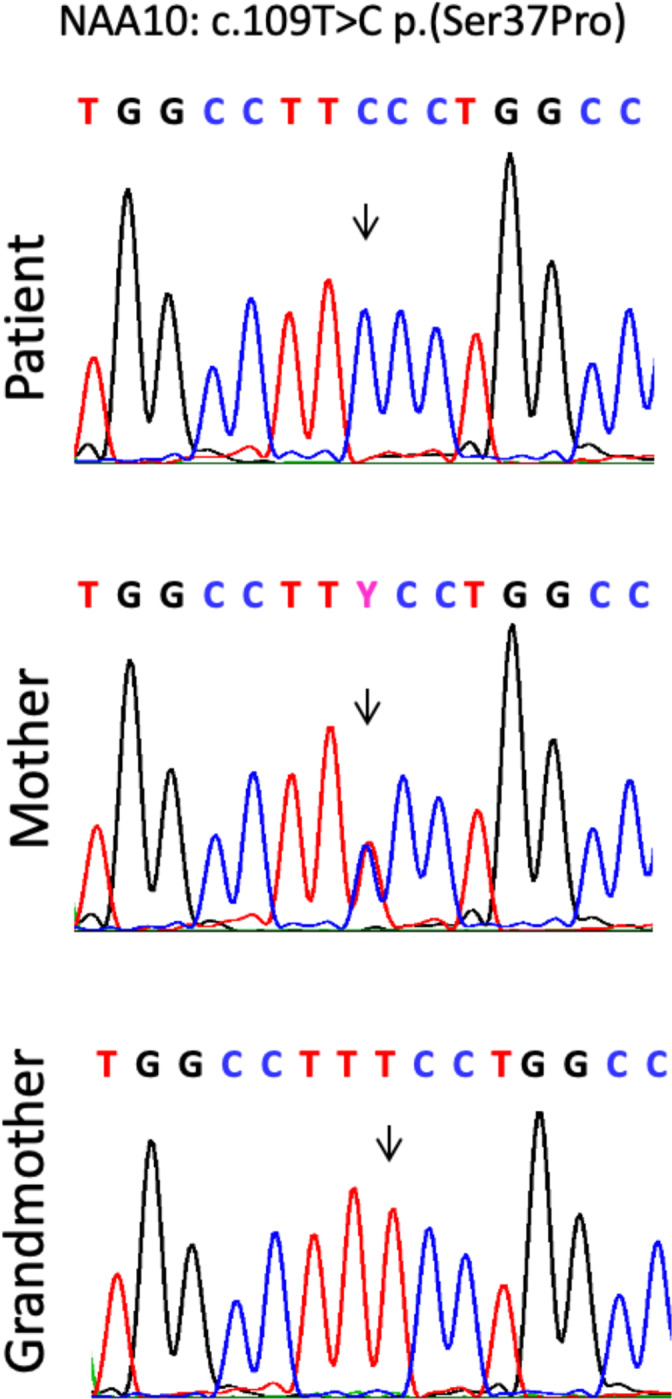
Partial electropherograms after Sanger sequencing of PCR amplifications of exon 2 of NAA10 in our patient, his mother, and grandmother

Chromosomal microarray analysis using an Affymetrix Cytoscan HD array with 2.65 million probes revealed normal results at a 20 kb resolution. Whole exome sequencing was performed using the Agilent SureSelectXT Clinical Research Exome Kit (V5) for capturing and paired‐end‐sequencing (HiSeq SBS Kit v4, 125 Fwd‐125 Rev, Q30‐value 90) on a HiSeq2500 sequencer (Illumina Inc.) resulting in 94.2% of the target region covered 20×. Coding regions and 12 flanking intronic basepairs were analyzed for potentially deleterious variants in known disease genes using annotations of SIFT, PhyloPhen, LRT, Mutation Taster, Mutation assessor, FATHMM, GERP and CADD as provided by the NextGene Viewer (Softgenetics) software. The only obviously pathogenic variant identified was the hemizygous variant NM_003491.2(NAA10):c.[109T>C] p.[(Ser37Pro)] chrX.hg19:g.[153199841T>C], rs387906701. The variant was confirmed by Sanger sequencing and also detected in the patient's mother but excluded in the maternal grandmother (see Figure 3). X‐Inactivation studies using the trinucleotide‐repeat of the AR‐gene as described (Lau et al. Am J Hum Genet [1997] 61:1353–1361) revealed ~100% skewing in the mother's blood probe. X‐inactivation studies at the SLITRK4 locus (Xq27.3) (Bertelsen et al. J Mol Diagn [2011] 13:537–540) showed a random X‐inactivation (48:52) in the blood probe of the maternal grandmother, who is not a carrier.

## 4 DISCUSSION

Our observation confirms the initial reports of Ogden syndrome as an X‐linked recessive early lethal condition in boys caused by the NAA10 variant Ser37Pro (Rope et al., 2011). Because the two previously described families shared a surrounding haplotype of not more than 1.5 cM, a founder variant was likely dismissed but could not be excluded (Rope et al., 2011). Since the variant occurred de novo in the boy's mother reported here, we can now unambiguously prove this variant to be recurrent. While carrier women of this variant are healthy with skewed X‐inactivation, the phenotype of affected boys is consistently severe (see Table 1) (Myklebust et al., 2015; Rope et al., 2011). The prematurely aged appearance with facial wrinkling, reduced subcutaneous adipose tissue and redundant skin is pathognomonic for this syndrome (Rope et al., 2011). All affected boys present the same facial anomalies such as prominent eyes, hypertelorism, downslanting palpebral fissures, broad philtrum, wide mouth and microretrognathia, as well as large, low‐set ears with abnormality of the pinna. As demonstrated in our case, some of these facial features are already recognizable on prenatal 3D ultrasound scans (see Figure 1). A variety of prenatal complications (e.g., IUGR, oligohydramnios, premature birth) were reported in almost all affected pregnancies. Growth parameters at birth were, except in our case, in the normal range, but in the further course weight, length and head circumference were below the 5th centile in five patients. Not all affected boys were described in great detail, but the typical clinical course was characterized by postnatal complications, which required in most of the times neonatal intensive care, particularly for respiratory distress and feeding difficulties. Muscular hypotonia, mainly of the trunk was present in several affected boys and some developed limb hypertonia. Cardiac anomalies were a persistent finding in more than half of the boys and included ventricular septal defect, atrial septal defect, pulmonary artery stenosis, and bicuspid aortic valve. Cardiac arrhythmias occurred in four and apneic episodes or desaturations in five patients. When specified, all boys presented with moderate to severe global developmental delay. Brain imaging, when undertaken, revealed various anomalies (i.e., enlarged ventricles, cerebral atrophy, immature myelination) in four boys and three boys developed seizures. Recurrent infections were described in four boys. Cryptorchidism/small testes were diagnosed in five patients and four had inguinal or umbilical hernias. While our patient showed persistent pupillary membrane, retinal pigmentary anomalies and an anomalous optic disc, except for a lagophthalmos no other eye anomalies were reported in the other affected boys (Rope et al., 2011). Scoliosis was diagnosed in our patient and in another boy (Rope et al., 2011). Our patient died at the age of 7 months, which confirms again the so far described lethal course of this syndrome. In our patient no cardiac arrhythmia was detected, but only two standard electrocardiograms were performed, which is not sufficient to exclude e.g. long QT‐syndrome. Premortal chest X‐rays revealed cardiomegaly and signs of obstructive bronchitis (see Figure 2), but since autopsy was denied the underlying cause of the respiratory failure was not fully understood. Clinical findings that were only present in our patient were the bilateral hip dislocation with an unilateral acetabular dysplasia, the ophthalmological anomalies and the adipose tissue mass (DD: teratoma) in the epipharynx.

**TABLE 1:** Clinical features of Ogden syndrome caused by the NAA10 Ser37Pro variant

	Our patient	Family1:II‐1 (Rope et al.)	Family1:II‐6 (Rope et al.)	Family1:III‐7 (Rope et al.)	Family1:III‐4 (Rope et al.)	Family1:III‐6 (Rope et al.)	Family2:II‐1 (Rope et al.)	Family2:III‐2 (Rope et al.)	Family2:III‐4 (Rope et al.)
Inheritance/X‐inactivation									
Maternally inherited	+	+	+	+	+	+	+	+	+
Skewing of X‐inactivation^a^	~100%	~90%	~90%	~100%	~100%	~100%	NA	NA	NA
Death at age	7 months	11.5 months	9.5 months	5.5 months	15 months	NA	8 months	11.5 months	16 months
Pregnancy									
IUGR	+	−	−	−	borderline	−	−	−	NA
Placental insufficiency	−	?	?	−	−	−	−	−	NA
Oligohydramnios	−	−	−	+	−	−	−	−	NA
Gestational diabetes	−	−	−	+	+	+	−	−	NA
Early labor contractions	−	−	−	−	+	−	−	−	NA
Decreased fetal movement	−	−	−	−	+	−	−	−	NA
Fetal supraventricular tachycardia	−	−	−	−	−	−	−	+	NA
Birth									
Gestational week	37	37 1/2	38	33	37 3/7	35 4/7	43	39.7	34
Weight	2580 g (P10‐25)	2140 g (P3‐10)	3065 g (P50)	1559 g (P10‐25)	2410 g (P10‐25)	2604 g (P50‐75)	3300 g (P25‐50)	2660 g (P5)	NA
Length	43 cm (<P3)	47 cm (P25‐50)	47 cm (P25‐50)	39 cm (P10)	44 cm (P3)	48 cm (P75)	51 cm (P50‐75)	46 cm (P5‐10)	NA
OFC	32.5 cm (P10)	32 cm (P10‐25)	33.75 cm (P50)	27 cm (P3‐10)	32 cm (P10‐25)	32.5 cm (P50‐75)	32 cm (P3)	32 cm (P5‐10)	NA
APGAR	9/10/10	1/1	4/6	4/8	6/7/9	4/9	6/8		NA
Perinatal course									
Respiratory distress	+	+	+	+	−	+	−		NA
Feeding difficulties	+	+	+	+	+	+	+		+
Hyperbilirubinemia	+	−	−	+	+	+	+		NA
Meconium aspiration	−	+	−	−	−	−	−	NA	NA
Thrombocytopenia	−	−	−	−	+	−	−		NA
Polycythemia	−	−	−	+	+	−	−		NA
Low cortisol	−	−	−	+	−	−	−		NA
Vasomotor instability	−	−	−	−	−	−	+		NA
Hypoglycemia	+	−	−	−	−	−	−		NA
Growth									
Short stature	+	NA	borderline	NA	−	+	+	+	NA
Microcephaly	+	+	−	NA	+	NA	+	+	NA
Decreased body weight	+	+	+	NA	+	NA	−	+	NA
Dysmorphism									
Aged appearance	+	+	+	+	+	NA	+	+	NA
Wrinkled forehead	+	+	+	+	+	(+)	+	+	NA
Downslanted palpebral fissures	+	+	+	+	+	+	+	−	NA
Prominent eyes	+	+	+	+	+	+	Bil. ptosis	+	NA
(Apparent)hypertelorismus	+	+	+	+	+	+	+	+	NA
Prominent nasal root	+	+	+	+	+	NA	+	+	NA
Wide nares	+	+	+	+	+	NA	+	+	NA
Broad philtrum	+	+	+	+	+	NA	+	+	NA
Wide mouth	+	(+)	+	(+)	+	NA	+	+	NA
Thin upper lip vermilion in frontal view	+	+	+	+	+	NA	+	+	NA
Microretrognathia	+	+	+	+	+	+	+	+	NA
Low‐set, large, posteriorly rotated ears	+	+	+	+	+	+	+	+	NA
Short neck	+	+	+	+	+	NA	(+)	(+)	NA
Neurologic abnormalities									
Developmental delay	Severe	Severe	Mod.–severe	NA	Severe	NA	+	+	NA
Seizures	+	−	−	−	−	−	+	+	NA
Muscular hypotonia	+	−	+	−	+	+	NA	−	NA
Hypertonia	+	−	+	−	−	−	NA	+	NA
Irritability	+	−	+	+	−	−	−	−	NA
Brain imaging abnormality									
Ventriculomegaly	+	+	−	NA	+	−	NA	NA	NA
Thin corpus callosum	+	−	−	NA	−	−	NA	NA	NA
Small pituitary gland	+	−	−	NA	+	−	NA	NA	NA
Cerebral atrophy	+	+	+	NA	−	−	NA	NA	NA
Delayed myelination	−	NA	NA	NA	+	NA	NA	NA	NA
Cardiac anomalies									
Atrial septal aneurysm	+	−	−	−	−	−	−	−	NA
Patent foramen ovale	+	−	−	+	+	−	−	−	NA
Patent ductus arteriosus	−	−	+	+	−	−	−	−	NA
Bicuspid aortic valve	+	−	−	−	−	+	−	−	NA
Periph. pulmonary stenosis	−	+	−	−	+	−	−	−	NA
Ventricle septal defect	−	?	−	−	+	−	−	−	NA
Pulmonary hypertension	−	−	−	−	−	+	−	−	NA
Left/right systolic dysfunction	−	−	−	+	−	−	−	−	NA
Dilated cardiomyopathy	?	?	−	+	−	−	−	−	NA
Cardiac arrhythmia	?	+	?	−	+	−	−	+	+
Apneic episodes	(+)	+	+	?	(+)	NA	NA	(+)	NA
Eye anomalies									
Pupillary membrane, enlarged optic disc, retinal hyperpigmentation	+	−	−	−	−	−	−	−	NA
Lagophthalmos	−	−	−	−	−	−	−	+	NA
Urogenital anomalies									
Cryptorchidism	−	+	+	+	+	+	−	−	NA
Enlarged abnormal kidneys	−	−	−	+	−	−	−	−	NA
Skeletal findings									
Large fontanels	+	+	+	+	+	+	−	NA	NA
Hip dislocation	+	−	−	−	−	−	+	NA	NA
Camptodactyly	+	−	−	−	−	−	−	NA	NA
Clinodactyly	−	+	−	−	−	−	−	NA	NA
Large great toes	+	+	−	−	−	+	−	NA	NA
Scoliosis	+	−	−	−	+	−	−	NA	NA
Frequent infections	+	−	+	NA	+	NA	NA	+	NA
Others									
Abnormal hearing	+	−	−	−	−	−	−	−	NA
Eczema	−	+	+	−	+	−	NA	+	NA
Adipose tissue mass (DD: teratoma)	+	−	−	−	−	−	−	−	NA
Umbilical hernia	−	−	+	−	−	−	−	−	NA
Inguinal hernia	−	−	−	+	−	+	−	+	NA
Cong. Lymphedema seq.	−	−	−	+	−	−	−	−	NA
Adrenomegaly	−	−	−	+	−	−	−	−	NA
Hirsutism	+	−	−	−	+	−	+	−	NA
Reduced subcutaneous fat	+	+	+	−	+	+	(+)	+	NA
Emesis/diarrhea	+	−	−	−	−	−	−	+	NA

Abbreviations: IUGR, intrauterine growth retardation; OFC, occipitofrontal circumference.

^a^ The degree of skewing for Family 1 of Rope et al. was estimated from fig. 3B of Myklebust et al. Hum Mol Genetics, 2015, Vol 24, No 7, pages 1956–1976; please note that there is a discrepancy between the values in the figure and the text.

Ogden syndrome was the first genetic syndrome linked to variant in a NAT (Rope et al., 2011). The NAA10 gene is located on the X‐chromosome (Xq28), has an universal expression pattern in human fetal and adult tissues, and encodes the catalytic subunit (NAA10) of NatA and the auxiliary subunit of NatE (Lee et al., 2018b; Pang et al., 2011; Park & Szostak, 1992; Tribioli et al., 1994). NATs catalyze the most common modification of eukaryotic proteins, namely the acetylation of proteins at their N‐terminus by transferring an acetyl moiety from acetyl coenzyme (Aksnes et al., 2016; Dörfel & Lyon, 2015; Friedmann & Marmorstein, 2013; Polevoda et al., 2009; Starheim et al., 2012). NatA is the major human NAT and is composed at least of the catalytic subunit Naa10, the auxiliary subunit Naa15, NAA50 and HYPK (Arnesen et al., 2005, 2006, 2010). NAA10 is mainly bound in complex with NAA15, ribosome‐ and non‐ribosome associated (Gautschi et al., 2003; Van Damme et al., 2011), yet there also exists a pool of non‐NAA15 bound NAA10 (Van Damme et al., 2011).

The Ser37Pro missense variant is localized in exon 2 and has been well studied in vitro and in vivo. The serine codon is highly conserved in eukaryotes and lies within the dimerization domain with Naa15 (see Table. 2) (Rope et al., 2011). The Ser37Pro variant showed decreased enzymatic activity in in vitro acetylation assays, reduced NatA‐complex formation and an impaired interaction with hNaa50, suggesting the lack of N‐terminal‐acetylation of a small number of proteins (Myklebust et al., 2015; Rope et al., 2011). Furthermore, S37P‐hTERT cells have reduced cell proliferation and dysregulation regarding cell–cell contact inhibition and migration (Myklebust et al., 2015). Lee et al. concluded that defects in DNA methylation and genomic imprinting may have as well a contributory role to the pathomechanism of Ogden syndrome, showing that the Ser37Pro variant disrupts ICR binding of Naa10 and Dnmt1 (Lee et al., 2018a).

**TABLE 2:** Phenotypes and genotypes of patients with NAA10‐related syndrome

	Ogden syndrome	“Ogden‐like syndrome”	Syndromic microopthalmia		(Non)specific ID	
	Rope et al. (2011); this report	Cheng et al. (2019)	Cheng et al. (2019), Esmailpour et al. (2014), Forrester et al. (2001), Graham et al. (1991), Johnston et al. (2019), Slavotinek et al. (2005)		Bader et al. (2020), Casey et al. (2015), Cheng et al. (2019), McTiernan et al. (2018), Popp et al. (2015), Rauch et al. (2012), Ree et al. (2019), Saunier et al. (2016), Shishido et al. (2020), Sidhu et al. (2017), Stove et al. (2018), Thevenon et al. (2016)	
					Male patients (*n* = 10)	Female patients (*n* = 37)
**NAA10 mutation** (number; gender)	Ser37Pro (9 males)	Asp10Gly (1 male)	c.471+2T>A (4 males) c.455_458del (1 male)	c.*39A>G (5 males) c.*40A>G (1 male) c.*43A>G (9 males)	Tyr43Ser Ile72Thr Arg83Cys Arg83His Arg116Trp c.455_458del	Leu11Arg Val111Gly His16Pro Arg116Trp Arg83Cys Leu121Val Ala87Ser Phe128Ile Ala104Asp Phe128Leu Val107Phe Met147Thr
**Inheritance** Maternally inherited De novo X‐inactivation in female carriers Female carrier phenotype	9/9 − 5/5 skewed, 3 not tested Not affected	− + − −	5/5 − NA Toe syndactyly, recurrent abortions	15/15 − 4/11 skewed Deviation of finger, pes planus, toe syndactyly	8/10 2/10 1/5 normal (4/5 NA) Not affected (4/5) Mild ID, arrhythmia, facial dysmorphism, skeletal findings (1/5)	1/37 (MGM) 35/37 (2NA) normal (6/37), skewed (3/37) −
**Abnormality of prenatal development or birth** (HP:0001197)	9/9	NA	4/4 (1/5 NA)	NA	3/10	7/24
**Perinatal course** Respiratory distress (HP:0002643) Failure to thrive (HP:0001508)	5/9 7/9	NA +	2/5 2/5	NA	1/10 3/10	NA 33/35
**Death in infancy** HP:0001522	9/9	+	−	−	1/10	NA
**Growth** Short stature (HP:0004322) Microcephaly (HP:0000252)	4/9 5/9	+ −	2/5 1/5	NA NA	4/10 1/10	23/35 15/35
Facial dysmorphism (HP:0001999)	8/9	+	5/5	NA	2/10 Rather unspecific (8/10)	30/36 (rather unspecific)
**Neurologic abnormalities** Neurodevelopmental delay/Intellectual disability (HP:0012758) Seizures (HP:0001250) Muscular hypotonia (HP:0001252) or Hypertonia (HP:0001276) Brain imaging abnormality (HP:0410263)	8/9 Severe 3/9 8/9 1/9 6/9	+ − + − +	5/5 Mild–severe 1/5 4/5 − 3/5	8/9 (c.*43A>G) mostly severe − − − −	10/10 Mild–severe 3/10 6/10 1/10 6/10	37/37 Mild–severe 12/28 30/31 − 23/28
**Behavioral abnormality** (HP:0000708)	−	−	5/5	10/12	4/10	33/36
**Abnormality of the CVS** Abnormal heart morphology (HP:0001627) Arrhythmia (HP:0011675)	5/9 2/9	+ −	3/5 −	NA	8/10 4/10	7/33 12/34
**Abnormality of the eye** (HP:0000478)	2/9	NA	5/5	11/12	3/10	25/32
**Abnormality of the genitourinary system** (HP:0000119)	4/9	NA	2/5	1/12	3/10	2/8
**Abnormality of the skeletal system** (HP:0000924)	7/9	+	5/5	4/12	6/10	16/30
**Abnormality of the gastrointestinal tract** HP:0011024	2/9	NA	5/5	–	3/10	7/26
**Abnormality of the ear** HP:0000598	8/9	+	3/5	2/12	1/10	8/22
**Hearing abnormality** HP:0000364		+				
**Recurrent infections** (HP:0002719)	8/9	NA	2/5	1/12	2/10	NA
**Functional studies of NAA10 variant**	Reduced catalytic capacity (60–80%) impaired NatA complex formation, reduced interaction between NatA and Naa50 (NatE)	Significantly lowered enzymatic activity, destabilization of the core of the catalytic subunit	**c.471+2T>A**: No detectable normally spliced NAA10 at RNA level, small amount of aberrant transcript and truncated protein, fibroblast cell growth defects, disrupted association with TSC2, significant dysregulation of genes involved in the retinoic acid and WNT signaling pathway	c.*39A>G and c.*43A>G Disruption of cleavage and polyadenylation, decrease of NAA10 RNA (~50%)	**His16Pro**: Impaired NatA complex formation, 4‐fold reduced catalytic activity of NatA complex **Tyr43Ser**: Significant decrease in catalytic activity, reduced stability **Ile72Thr**: 75% reduction in catalytic activity toward the in vitro monomeric NAA10 substrate (no reduction in catalytic activity NatA substrate) **Arg83Cys**: Clear reduction (~60%) of the catalytic activity **Val107Phe**: Nearly abolished enzymatic activity **Val111Gly**: Decreased stability, decreased monomeric catalytic activity, NatA catalytic activity remained unchanged **Arg116Trp**: Very mild reduction in the catalytic activity **Phe128Ile**: Near loss (>90%) of the catalytic activity, reduced stability	

Abbreviations: CVS, cardiovascular system; ID, intellectual disability; MGM, maternal germline mosaicism; NA, not available; WNT, wingless‐related integration site.

**FIGURE 4:**
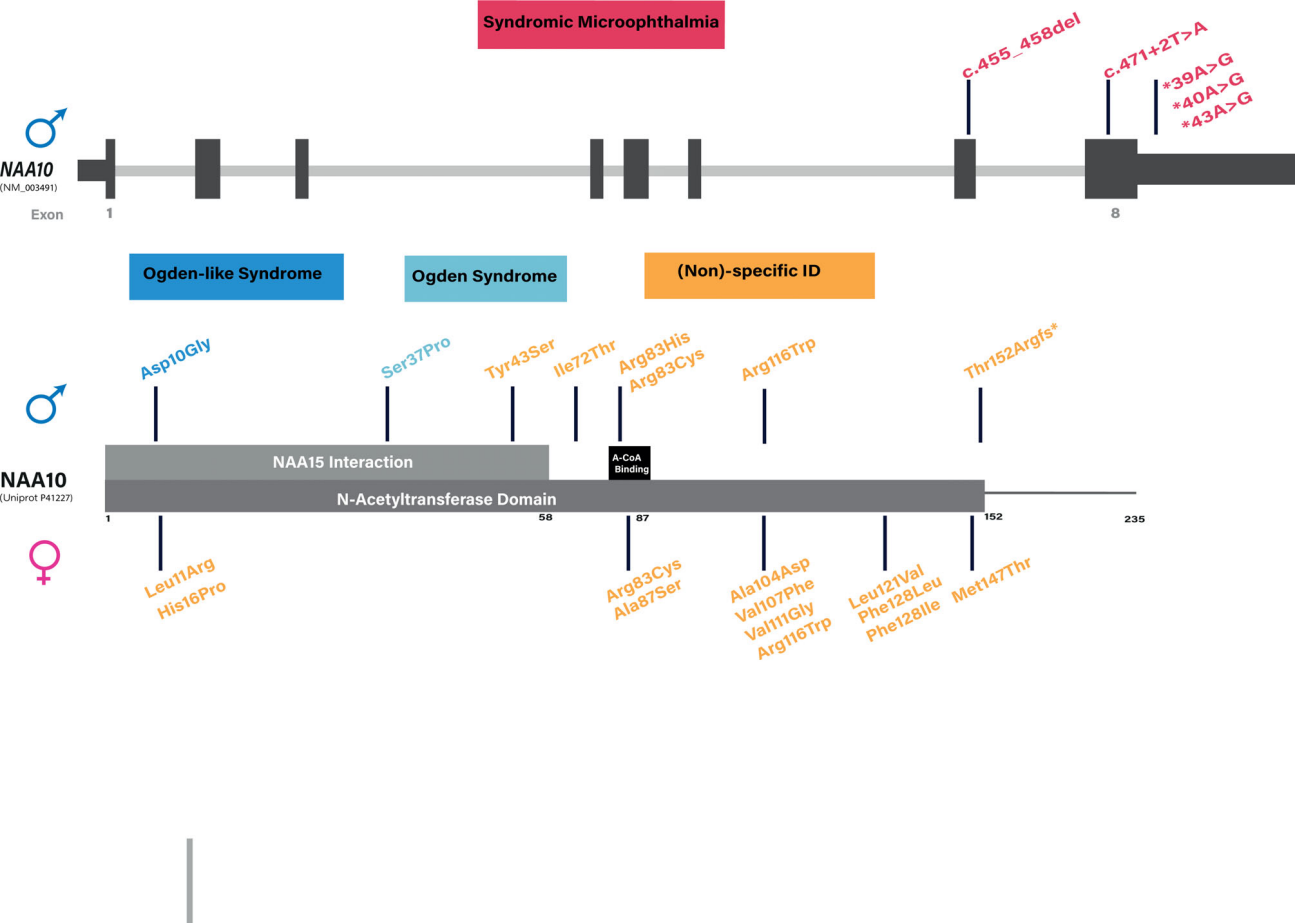
Schematic representation of the genomic structure plus the functional domains of the human NAA10 gene with representation of the genotype–phenotype correlation of the so far described NAA10 pathogenic variants in males (above NAA10 and NAA10) and in affected females index cases (below NAA10). The genomic structure is based on isoform 1, encoded by transcript NM_003491.3. The domain structure corresponds to the following Uniprot identifiers P41227

No other case of Ogden syndrome has been published since it was first described in 2011. But in 2018, Cheng et al. identified a de novo hemizygous missense variant Arg10Gly in a 4.5 month‐old boy with an Ogden syndrome‐like phenotype with remarkably similar facial features and a severe, early lethal clinical course (Cheng et al., 2020). Functional characterization showed that this variant has similar cellular effects as the Ser37Pro variant, explaining the severe lethal phenotype (Cheng et al., 2020). Wu and Lyon suggested that the term Ogden syndrome should be reserved for male infants with the fatal neonatal presentation (Wu & Lyon, 2018). Although the boy had a fatal neonatal outcome the authors Cheng and Lyon did not label this case as Ogden syndrome, maybe because the NAA10 variant occurred de novo in this case and it was not totally clear if it was X‐linked recessive (Cheng et al., 2020). Thus, more detailed descriptions of the fatal NAA10‐related disorder are needed to fully understand the genetic and clinical heterogeneity of Ogden‐syndrome. After uncovering Ogden syndrome in 2011, reports of different nonlethal NAA10 variants in males as well as mostly de novo variants in affected female index cases with nonspecific ID have been published, expanding the clinical spectrum and elucidating the underlying complexity of NAA10 deficiency (see Figure 4). An excellent overview of these overlapping yet distinct disorders associated with pathogenic variants in the NAA10 gene was published by Wu and Lyon, who recommend that these diseases should be referred to more broadly as NAA10‐related syndrome, given the enormous phenotypic variability (Wu & Lyon, 2018). A comparative synopsis of the different clinical presentations is depicted in Table 2, showing on the one hand the large heterogeneity and on the other hand the consistency of the phenotype of NAA10‐related syndrome, for example, developmental delay ranging from mild to severe, muscular hyptonia, skeletal and growth abnormalities, cardiac anomalies and recurrent infections. The cause of the wide‐ranging phenotypic differences, the disease progression and severity among different NAA10 variants, especially in boys, is still a topic of debate. In 2012, Rauch et al. showed first that another NAA10 de novo missense variant Arg116Trp can also cause a nonlethal phenotype in a male patient with nonspecific severe ID (Rauch et al., 2012). In vitro studies showed that this variant has a significant but rather small reduction in the catalytic activity (15%) in comparison to the Ser37Pro variant, a plausible explanation for the mild phenotype in the boy (Popp et al., 2015). However, in 2015, Casey et al. argued that the relation between Naa10 variants and disease phenotypes is more complex and that the in vitro catalytic activity in itself is not always sufficient in explaining phenotypes observed in patients (Casey et al., 2015). The group identified a maternally inherited missense variant Tyr43Ser in the NAA10 gene, resulting in a severely impaired catalytic activity compared to the Ser37Pro variant, in two affected brothers with intellectual disability, cardiac arrhythmia including long QT, dysmorphic and skeletal features (syndromic ID) (Casey et al., 2015). This assumption was supported by the group of Ree et al., who identified a de novo NAA10 missense variant Arg83His, leading to a greatly reduced catalytic activity due to impaired Ac‐CoA binding in two unrelated boys with intellectual disability, developmental delay, ADHD like behavior, very limited speech and cardiac abnormalities (Ree et al., 2019). An additional possible pathomechanism, referring to the different substrate specificity of NAA10, was proposed by Stove et al., who published a previously undescribed maternally inherited NAA10 variant Ile72Thr in three boys from two unrelated families with a milder phenotypic spectrum consisting of developmental delay, intellectual disability, cardiac arrhythmia and hypertrophic cardiomyopathy (Stove et al., 2018). Functional studies of this variant revealed an increased turnover rate when expressed in human cell lines, a 75% reduction in catalytic activity toward the in vitro monomeric NAA10 substrate, but surprisingly no reduction in catalytic activity when tested for the NatA substrate, leading to the assumption that this Ile72Thr is protected from degradation and destabilization by forming a functional NatA complex (Stove et al., 2018). In summary these functional studies of NAA10 missense variants, demonstrating a variable effect on the ability of NAA10 to acetylate substrates, are supporting the hypothesis that the level of acetylation dysfunction and affected substrates is an important underlying pathomechanism with a possible correlation toward the clinical phenotype (Johnston et al., 2019).

Compared to missense variants, alterations in the NAA10 gene which result in a (partial) loss of protein function due to reduced mRNA levels can lead to syndromic microphthalmia/anophthalmia in boys (Esmailpour et al., 2014; Johnston et al., 2019; Shishido et al., 2020). Complete loss‐of‐function of NAA10, however, is thought to be lethal in males (Popp et al., 2015).

The majority of variants in male patients were maternally inherited and most of the female carriers were healthy or had only minor features (see Table 2). But as already mentioned, female index patients with pathogenic, apart from one maternal mosaic variant merely de novo missense variants in the NAA10 gene have been equally identified over the time. The phenotype in females with NAA10‐related N‐terminal‐acetyltransferase deficiency is among others characterized by moderate to severe developmental delay and ID with no or very limited speech, postnatal growth failure, microcephaly, seizures, different skeletal, brain, and organ anomalies and cardiac arrhythmias (see Table 2) (Bader et al., 2020; Cheng et al., 2020; McTiernan et al., 2018; Popp et al., 2015; Saunier et al., 2016; Sidhu et al., 2017; Thevenon et al., 2016).

X‐chromosome inactivation studies in affected females, when undertaken, were not conclusive, showing either random or skewed X‐inactivation. Studies of acetylation activity revealed severely abolished activity in most but not all of these de novo variants causing a severe phenotype in females (Cheng et al., 2020; Popp et al., 2015; Saunier et al., 2016).

Ogden syndrome was the first genetic disorder which contributed highly to the research and decipherment of the fascinating role of N‐terminal acetylation in development and disease. With the identification of different NAA10 alterations, we slowly gain insight into its complexity and are starting to unravel the contributing factors and mechanism underlying this broad clinical spectrum of NAA10‐related disorders.

## 5 CONCLUSION

In summary we presented the ninth case of a boy with Ogden syndrome who was diagnosed in Zurich, Switzerland more than 5000 miles away from the hometown of the original family in Utah, USA. We could confirm the consistency of the clinical phenotype and recurrence of this rare lethal X‐linked recessive disorder caused by the NAA10 Ser37Pro variant. Our patient exhibited the full phenotypic spectrum of Ogden syndrome (Aksnes et al., 2016), which is a highly recognizable syndrome, in particular due to the remarkable similar facial gestalt of affected boys. We further delineated the evolving phenotype of this condition, additionally presented new clinical findings and reflected shortly on the extensive phenotypic variability that is linked to different variants in the NAA10 gene.

## CONFLICT OF INTEREST

The authors declare no conflicts of interest.

## AUTHOR CONTRIBUTIONS

Laura Gogoll wrote the article with support and supervision of Katharina Steindl and Anita Rauch. Katharina Steindl and Laura Gogoll contributed as well to the clinical evaluation of the patient. Alessandra Baumer and Pascal Joset performed the genetic analysis of the patient. Christina Gehrt‐Kahlert and Boris Tutschek provided additional clinical data and pictures. All authors reviewed the article and contributed to its content.

## INFORMED CONSENT STATEMENT

Written informed consent has been obtained from the family.

## Data Availability

Data sharing is not applicable to this article as no new data were created or analyzed in this study.
